# SAMHD1 in cancer: curse or cure?

**DOI:** 10.1007/s00109-021-02131-w

**Published:** 2021-09-04

**Authors:** Kerstin Schott, Catharina Majer, Alla Bulashevska, Liam Childs, Mirko H. H. Schmidt, Krishnaraj Rajalingam, Markus Munder, Renate König

**Affiliations:** 1 Host-Pathogen Interactions, Paul-Ehrlich-Institut, Langen, Germany; 2 Institute of Anatomy, Medical Faculty Carl Gustav Carus, Technische Universität Dresden School of Medicine, Dresden, Germany; 3 Cell Biology Unit, University Medical Center of the Johannes Gutenberg University Mainz, Mainz, Germany; 4 University Cancer Center Mainz, University Medical Center Mainz, Mainz, Germany; 5 Third Department of Medicine, University Medical Center, Johannes Gutenberg University, Mainz, Germany

**Keywords:** SAMHD1, Cancer development, dNTP regulation, Cellular functions of SAMHD1, Mutations in SAMHD1

## Abstract

Human sterile α motif and HD domain-containing protein 1 (SAMHD1), originally described as the major cellular deoxyribonucleoside triphosphate triphosphohydrolase (dNTPase) balancing the intracellular deoxynucleotide (dNTP) pool, has come recently into focus of cancer research. As outlined in this review, SAMHD1 has been reported to be mutated in a variety of cancer types and the expression of SAMHD1 is dysregulated in many cancers. Therefore, SAMHD1 is regarded as a tumor suppressor in certain tumors. Moreover, it has been proposed that SAMHD1 might fulfill the requirements of a driver gene in tumor development or might promote a so-called mutator phenotype. Besides its role as a dNTPase, several novel cellular functions of SAMHD1 have come to light only recently, including a role as negative regulator of innate immune responses and as facilitator of DNA end resection during DNA replication and repair. Therefore, SAMHD1 can be placed at the crossroads of various cellular processes. The present review summarizes the negative role of SAMHD1 in chemotherapy sensitivity, highlights reported SAMHD1 mutations found in various cancer types, and aims to discuss functional consequences as well as underlying mechanisms of SAMHD1 dysregulation potentially involved in cancer development.

**Supplementary Information:**

The online version contains supplementary material available at 10.1007/s00109-021-02131-w.

## Introduction

**Fig. 1:**
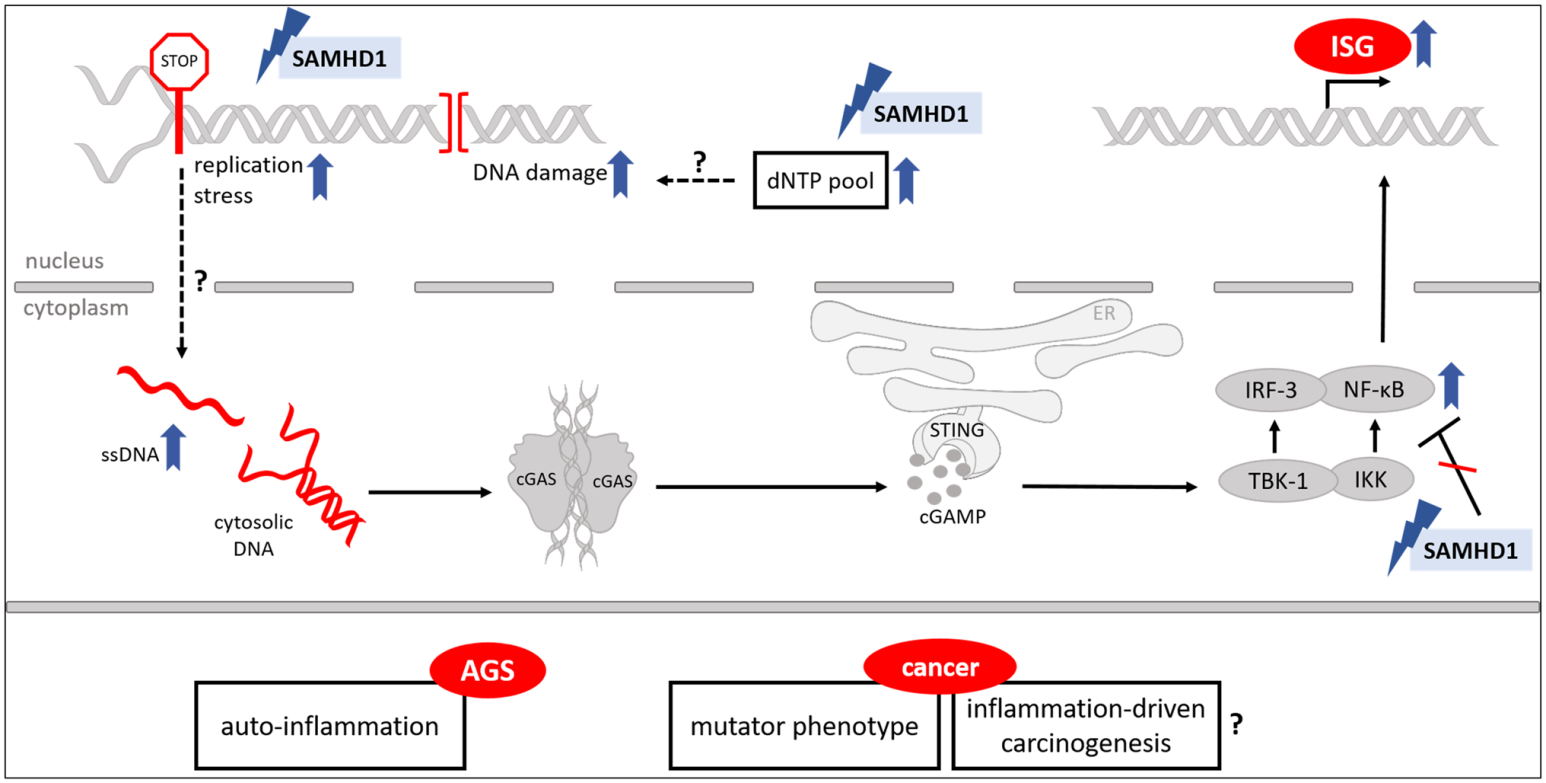
SAMHD1, its functions, and implications for AGS and cancer Cellular functions of SAMHD1 and functional consequences for mutated/dysfunctional or downregulated SAMHD1 are depicted. Mutated SAMHD1 might lead to displacement of ssDNA into the cytoplasm, where it can be detected by intracellular DNA sensors like cGAS. cGAS then produces cyclic guanosine monophosphate-adenosine monophosphate (cGAMP) to activate STING which in turn activates interferon regulatory factor 3 (IRF3) and the NF-κB pathways through the kinases TANK-binding kinase 1 (TBK1) and IκB kinase (IKK), thus inducing an IFN response. Consequences of dysfunctional SAMHD1 on AGS and cancer are displayed in the lower part of the figure. Unclear relations and consequences are indicated by question marks. Image created with Servier Medical Art (https://smart.servier.com/)

Human sterile α motif and HD domain-containing protein 1 (SAMHD1) was first described to be the major cellular deoxyribonucleoside triphosphate triphosphohydrolase (dNTPase) and to be crucial for controlling cellular deoxynucleotide (dNTP) levels [1, 2]. At present, the role of SAMHD1 in a variety of cancer types has been studied and will be highlighted in this review. Besides its role as a dNTPase, several novel functions have been attributed to SAMHD1. These include a direct role of SAMHD1 as a negative regulator of innate immunity [3], and a role in promoting the end resection process during DNA repair by recruitment of CtBP-interacting protein (CtIP) endonuclease to DNA damage sites [4] and during DNA replication by resolving stalled replication forks through recruitment of MRE11 Homolog, Double Strand Break Repair Nuclease (MRE11) and stimulating its exonuclease activity [5] (Fig. 1).

Furthermore, described mutations in SAMHD1 can cause the hereditary encephalopathy and interferonopathy Aicardi-Goutières syndrome (AGS) [6]. The exact mechanism of how mutated or inactive SAMHD1 triggers a type I interferon-mediated response is not yet clear. It is hypothesized to result from accumulation of self-derived nucleic acids, which trigger this response (Fig. 1) [7]. The source of the endogenous nucleic acids is as yet unclear; however, the various functions of SAMHD1, when inactive, could promote their accumulation and possibly lead to tumor-promoting inflammation (Fig. 1). It is known that unresolved DNA damage could lead to release of aberrant DNA into the cytosol, thus stimulating the cytosolic DNA sensor cyclic GMP-AMP Synthase (cGAS) and its adaptor Stimulator of Interferon Genes (STING) [8]. Therefore, mutations in SAMHD1 affecting DNA repair may lead to subsequent interferon (IFN) activation. Furthermore, mutations impairing end resection processes during DNA replication could lead to accumulation of aberrant DNA dislocated into the cytoplasm. Coquel et al. proposed that this aberrant DNA could activate innate immune signaling by cGAS/STING [5]. Also, mutations that impair the negative role of SAMHD1 within the nuclear factor kappa-light-chain-enhancer of activated B cells (NF-κB) and Interferon regulatory factor 7 (IRF7) pathway might also come into play [3].

SAMHD1 is placed at the crossroads of various cellular processes, including cell cycle progression and proliferation. Whether SAMHD1 deficiency affects cell proliferation, however, is still under debate. In transformed cells, contrasting results were reported: SAMHD1 deficiency was shown to lead to reduced cell growth and altered replication dynamics [5, 9]. On the other hand, it was shown that SAMHD1 deficiency led to stimulation of cell proliferation and reduced spontaneous apoptosis induction [10]. Moreover, mutated or downregulated SAMHD1 could lead to improperly regulated nucleotide metabolism as well as malfunctioning DNA replication and repair processes which will potentially lead to genomic instability and accumulation of mutations (Fig. 1). Together with resistance to apoptosis [10], SAMHD1 is involved in several cellular processes which are important hallmarks of cancer when dysregulated, as defined and summarized by Hanahan and Weinberg (2011). SAMHD1 might influence a variety of hallmarks, potentially including tumor-promoting inflammation as an enabling characteristic in neoplastic disease [11]. Therefore its role in the development in different cancer types remains to be firmly investigated. Furthermore, it will be important to dissect the influence of the dNTPase function of SAMHD1 on the effects observed in cancer cells, or whether additional functions contribute to tumorigenesis. This will enable a better understanding of SAMHD1 as a target for cancer therapy.

## SAMHD1 mutations reported in various cancer types

**Fig. 2:**
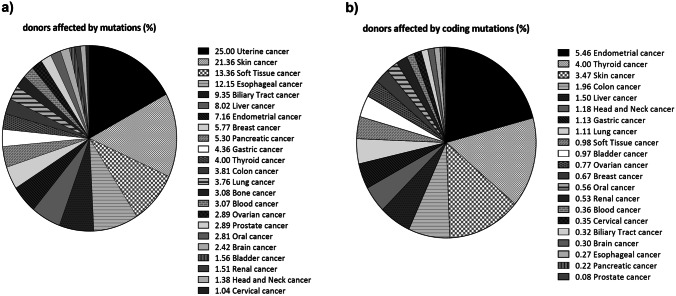
Donors affected by mutations in SAMHD1 per cancer type The distribution of all (**a**) and only coding somatic mutations (**b**) across the 20 most prevalent ICGC cancer studies is represented. The ICGC data portal offers clinical and analyzed data representing 81 cancer type datasets available from the ICGC Data Coordination Center for Release 28 (human genome hg19/GRCh37), processed as of March 27, 2019. We used open-access simple somatic mutations (SSM) calls. These include single and multiple base substitutions, and small (≤ 200 bp) insertions and deletions that appear in the tumor tissue, but not in the normal control tissues. The figure legends in **a** and **b** depict all surveyed cancers that are included in the pie charts along with the calculated percentage (%) of donors affected by each cancer type

To investigate the mutation spectrum of SAMHD1 in cancer, we queried the International Cancer Genome Consortium (ICGC) database [12]. We found 1542 mutations of SAMHD1 affecting 957 donors across 65 cancer projects. Figure 2a illustrates the extent to which mutations occur in each cancer type. A high prevalence of mutations in Fig. 2a is due to intronic mutations and mutations within the 5′ and 3′ UTR. As we were interested in mutations likely to change the protein function, we calculated the percentage of donors affected per cancer type for only coding mutations (Fig. 2b). Missense mutations are more represented than all others among the coding mutations (Supplemental Table 1). The five most prevalent cancer types affected by coding mutations are endometrial, thyroid, skin, colon and liver cancer (Fig. 2b). Additionally, SAMHD1 was identified to be recurrently mutated in certain hematological malignancies (Fig. 2, “blood” cancer) and analyzed in detail as outlined in the next paragraph.

Chronic lymphocytic leukemia (CLL), the most frequent type of leukemia in adults, is characterized by heterogeneous and constantly changing cell populations, leading to complications like treatment relapse or resistance to chemotherapy [13]. Using whole-genome sequencing (WGS), Schuh et al. monitored shifts in tumor subclone populations in three patients over the course of CLL treatment. In one patient, a somatic mutation in *SAMHD1* (c.1635 T > A; aa exchange: F545L) was identified in the founder subclone and present during all time points examined, indicating this mutation to be an early, potentially driving event [14]. Underscoring the role of *SAMHD1* mutations in CLL, a patient carrying a homozygous germ-line mutation in *SAMHD1* (c.1609-1G > C) was described in a subsequent study who was diagnosed with CLL at only 24 years of age — with no other acquired mutations or chromosomal lesions detectable known to be recurrently found in CLL [15]. Further analysis of clinical trial samples revealed that *SAMHD1* mutations were present in 3% (pretreatment group) to 11% (relapsed/refractory group) of CLL patients [15]. Additionally, pre-existing subclones with mutations in *SAMHD1* were enriched after therapy in another cohort of relapsed/refractory CLL (rCLL) patients, identifying genomic changes in *SAMHD1* as possible drivers of relapse [16]. The authors even hypothesize that *SAMHD1* mutations might contribute, to a certain extent, to CLL treatment resistance in vivo [16]. Furthermore, rCLL patients with > 1 gene mutation in nine recurrently affected genes (including *SAMHD1* + either *ATM*/*SF3B1*/*NOTCH1*) showed significantly poorer outcome in terms of overall survival (OS) compared to patients with no or only one mutation [17]. Additionally, it is important to further understand mutational differences in CLL subtypes, which differ in the abundance of somatic hypermutations affecting the Ig variable heavy-chain locus (IgHV^mut^ and IgHV^unmut^), since IgHV^unmut^ patients display a more aggressive form of CLL with poorer OS compared to IgHV^mut^ patients [18]. Burns et al. showed that mutations in the coding region and regulatory elements of *SAMHD1*, as well as in other known and potential CLL driver genes, were predominant in IgHV^unmut^ CLL patients [19]. In future studies, it will be important to understand the effects of *SAMHD1* mutations, especially in combination with other mutated genes, on the clinical outcome of different CLL patient groups in more detail.

As described, initial indications that *SAMHD1* mutations might be involved in cancer development/progression were derived from the occurrence of early-onset CLL in an AGS patient [15]. By now, the case study of a patient initially diagnosed with SAMS (stroke, aneurysm, moyamoya, and stenosis) association, attributed to a homozygous mutation (c.1411-2A > G) affecting a splice acceptor site in *SAMHD1* [20], was described who later developed a CD8^+^ epidermotropic cutaneous T-cell lymphoma (CTLC) at the age of 29 [21].

Apart from being recurrently mutated in rCLL, *SAMHD1* mutations were also found in 18% of patients with T-cell prolymphocytic leukemia (T-PLL) [22]. In this study, *SAMHD1* was identified as the second most frequently mutated gene, after *ATM*, and several mutations even presented as homozygous or hemizygous [22]. In T-PLL, some *SAMHD1* mutations resulted in reduced mRNA expression; however, protein expression was reduced or even absent in all samples from T-PLL patients with *SAMHD1* mutations [22]. Only recently, mutations in *SAMHD1* were also detected in 7.1% (13/182) of mantle cell lymphoma (MCL) patients selected from the MCL Younger and Elderly trials; of note, both cohorts only included previously untreated patients [23]. MCL is an rare subtype of B-cell non-Hodgkin lymphoma, which shows an aggressive course of disease and is still considered incurable [24]. In mutated MCL cases, SAMHD1 protein expression decreased compared to SAMHD1-unmutated patients, although the difference did not reach significance [23]. However, the mutation status of *SAMHD1* had no significant influence on failure-free survival (FFS) of MCL patients [23].

By now, a single case of an extremely rare low-grade B-cell lymphoma with IRF4 rearrangement with concomitant mutation in *SAMHD1* (c.G692A; leading to premature a stop codon, W231X) was described [25]. Probably, this early stop-gain mutation in *SAMHD1* will lead to reduced protein expression, which was not assessed in the report by Zhou et al. In this patient, further missense and frameshift mutations were detected in the genes *KMT2D*, *BTG1*, *PTEN*, and *BAX* [25].

As *SAMHD1* mutations were identified in several hematological malignancies, future efforts will be important to investigate whether *SAMHD1* mutations can be found in other tumors of hematopoietic and lymphoid origin and to further pinpoint which exact amino acid residues in SAMHD1 are affected, in order to investigate their roles in SAMHD1’s diverse cellular functions. Importantly, changes in SAMHD1 protein functions and/or expression levels due to deleterious mutations can have significant influence on the therapeutic outcome of specific cancer treatments (see last section of this review).

Apart from hematological malignancies, first studies on the role of *SAMHD1* mutations in the development and progression of solid tumors were conducted only in recent years. Using the colorectal cancer (CRC) data set deposited in The Cancer Genome Atlas (TCGA), Rentoft et al. identified eight different, nonsynonymous mutations in the coding region of *SAMHD1*; here, the amount of mutations in *SAMHD1* was higher than expected by chance [26]. Interestingly, all eight mutations were found in hypermutated colon cancers (> 12 mutations per 10^6^ bases) — with six of these tumors carrying additional mutations in genes important for mismatch repair (MMR). In *S. cerevisiae*, even a minor elevation in dNTP concentrations, in combination with mutated MMR genes, can lead to reduced DNA replication fidelity and, as a consequence, increased mutation rates [26]. It will be of great interest to study a possible interplay of SAMDH1 and MMR defects in mammalian cells, in general, and specifically in different tumor types. In vitro characterization of selected CRC-associated SAMHD1 mutants (V133I, A338T, R366H, D497Y) (Table 1) revealed that, indeed, all of them showed reduced or even completely abolished dNTPase activity compared to wild-type (wt) SAMHD1 [26]. In addition, some mutations (like R366H) did not influence hydrolysis of individual dNTPs to the same extent (2.5- to 11-fold for deoxyadenosine triphosphate/deoxycytidine triphosphate/deoxythymidine triphosphate (dATP/dCTP/dTTP), almost no effect on deoxyguanosine triphosphate (dGTP)), indicating that not only absolute, but also relative dNTP levels could be influenced by *SAMHD1* mutations [26]. Using hemizygous *SAMHD1*^+/−^ mouse embryos, Rentoft et al. could show that inactivation of only one SAMHD1 allele leads to elevation of cellular dNTPs. Consequently, the authors speculated that heterozygous, inactivating *SAMHD1* mutations would also disturb dNTP pools in vivo. However, in future studies, the exact impact of (heterozygous) *SAMHD1* mutations on dNTP levels/balance and/or mutation rates needs to be addressed using primary CRC patient samples.

**Table 1:** Selection of amino-acid positions/mutations in SAMHD1 in various cancers or AGS

**Amino acid position**	**Described function/structural significance**	**Mutation**	**Associated disease**	**Functional consequence of mutation**
H123	Primary allosteric dGTP/GTP-binding site [49]	120_123del	AGS [6]	Reduced LINE-1 restriction [99]
		H123P	AGS [6, 43]	Subcellular localization affected, partially nuclear and cytosolic [37]; reduced LINE-1 restriction [99]; no oligomerization [37]
		H123Y	Skin melanoma [ICGC]	
V133		V133I	Colon adenocarcinoma [26]	Reduced dNTPase activity [26]
D137	Primary allosteric dGTP/GTP-binding site [1, 100]	D137A	-	Loss of dNTPase activity and HIV-1 restriction [1, 101]
		D137H	Uterine corpus endometrial carcinoma [ICGC]	
		D137N	Colon adenocarcinoma [ICGC]	
Q142	Primary allosteric dGTP/GTP-binding site [1, 100]	Q142A	-	Reduced dNTPase activity [1]
		Q142X	Esophageal adenocarcinoma [ICGC]	
R143	Points from primary allosteric dGTP/GTP-binding site to the rear of the active site [50]	R143C	AGS [6, 43]; uterine corpus endometrial carcinoma [ICGC]	Subcellular localization affected, partially nuclear and cytosolic [37]; no oligomerization [37]; loss of HIV-1 restriction [50]
		R143H	AGS [6, 43]; liver hepatocellular carcinoma [ICGC]	Subcellular localization affected, partially nuclear and cytosolic [37]; reduced LINE-1 restriction [99]; no oligomerization [37]
		R143X	AGS [43]	
R145	Primary allosteric dGTP/GTP-binding site [1]	R145A	-	Loss of dNTPase activity [1, 50]
		R145Q	AGS [6, 43]; CLL [15]; colon adenocarcinoma [ICGC]	Subcellular localization affected, partially nuclear and cytosolic [43]; reduced LINE-1 [99] and loss of HIV-1 restriction [50]; no oligomerization [37]; loss of dNTPase activity [1]
		R145X	AGS [6, 43]; CLL [15]; pancreatic adenocarcinoma [ICGC]; uterine corpus endometrial carcinoma [ICGC]	Subcellular localization affected, partially nuclear and cytosolic [43]
R164	Forms salt bridge with phosphate group of dNTP bound to active site [1, 101]	R164A	-	Loss of dNTPase activity [1, 50]
		R164Q	Pancreatic adenocarcinoma [ICGC]; uterine corpus endometrial carcinoma [ICGC]	
		R164X	AGS [43]	Subcellular localization affected, partially nuclear and cytosolic [43]
H167	Coordinates cation in active site [1, 101]	H167Y	AGS [43]	Subcellular localization affected, partially nuclear and cytosolic [37]; reduced LINE-1 and HIV-1 restriction [37, 99]; no oligomerization [37]; reduced/loss of dNTPase activity [37]
I201		I201N	AGS [6, 43]; CLL [15]	Subcellular localization affected, mainly cytosolic [43] /partially nuclear and cytosolic [37]; reduced LINE-1 and HIV-1 restriction [37, 99]; reduced oligomerization [37]; reduced/loss of dNTPase activity [37]
H206	Coordinates cation in active site [1, 101]	H206A/D207A	-	Loss of dNTPase activity [1]; proficient in HR and DNA end resection [4]
		H206R	CLL [15]	
		H206Y	Skin cutaneous melanoma [ICGC]	
D207	Coordinates cation in active site [1, 101]	H206A/D207A	-	Loss of dNTPase activity [1]; proficient in HR and DNA end resection [4]
		D207Y	Colon adenocarcinoma [26]	
G209		G209C	Lung squamous cell carcinoma [ICGC]	
		G209S	AGS [6, 43]	Subcellular localization affected, partially nuclear and cytosolic [43]; reduced LINE-1 restriction [99], but restrictive against HIV-1 [37]; normal oligomerization and dNTPase activity [37]
F217		F217C	AGS [43]	Subcellular localization affected, partially nuclear and cytosolic [37]; reduced HIV-1 restriction [37]; no oligomerization [37]; reduced/loss of dNTPase activity [37]
		F217X	AGS [43]	
R226		R226G	AGS [43]	Subcellular localization affected, partially nuclear and cytosolic [37]; reduced HIV-1 restriction [37]; strongly reduced oligomerization [37]; reduced/loss of dNTPase activity [37]
		R226H	Colon adenocarcinoma [26]	
H233	Forms salt bridge with phosphate group of dNTP bound to active site [1]	H233A	-	Reduced dNTPase activity [1]; loss of HIV-1 restriction [50]
		H233Y	Liver hepatocellular carcinoma [ICGC]	
M254		M254I	CLL [44, 102, 103]; skin cutaneous melanoma [ICGC]	
		M254V	AGS [6, 43]	Subcellular localization affected, mainly cytosolic [43]/partially nuclear and cytosolic [37]; reduced LINE-1 and HIV-1 restriction [37, 99]; normal oligomerization [37]; reduced/loss of dNTPase activity [37]
R290		R290C	CLL [15]	
		R290H	AGS [43]; solon adenocarcinoma [ICGC]; stomach adenocarcinoma [ICGC]	Subcellular localization affected, partially nuclear and cytosolic [37]; reduced LINE-1 restriction [99]; no oligomerization [37]
		R290S	Liver hepatocellular carcinoma [ICGC]	
		R290X	CLL [44, 102]	
R305		R305A	Rectum adenocarcinoma [ICGC]	Loss of dNTPase activity and loss of ssDNA cleavage activity [45]
D311	Coordinates cation in active site [1]	D311A	-	Subcellular localization not affected, mainly nuclear [37]; reduced HIV-1 restriction [37]; normal oligomerization [37]; reduced/loss of dNTPase activity [1, 37]
		D311E	Breast invasive carcinoma [ICGC]	
R333	Secondary allosteric dNTP-binding site [49, 101]	R333C	Uterine corpus endometrial carcinoma [ICGC]	
		R333E	-	Reduced tetramer formation and dNTPase activity [101]
		R333H	AGS [ICGC]; breast invasive carcinoma [ICGC]; pediatric brain tumor [ICGC]; uterine corpus endometrial carcinoma [ICGC]	
		R333S	Breast invasive carcinoma [ICGC]	
A338		A338T	Colon adenocarcinoma [26]	Reduced dNTPase activity [26]
		A338V	Colon adenocarcinoma [ICGC]	
Y360	ssDNA binding [55]	Y360H	CLL [103]	
H364	Forms hydrogen bonds at dimer-dimer interface [53]; ssDNA binding [55]	H364K	-	Reduced tetramer formation and dNTPase activity [53]
		H364Q	Liver hepatocellular carcinoma [ICGC]	
		H364Y	Lung adenocarcinoma [ICGC]	
R366	Interacts with dNTP bound to active site [101]	R366C	CLL [17]	
		R366H	Colon adenocarcinoma [26]	Reduced dNTPase activity [26]
L369	Located at tetramer interface [49]	L369S	AGS [6, 43]	Subcellular localization affected, partially nuclear and cytosolic [37, 43]; reduced HIV-1 restriction [37]; reduced oligomerization [37]; reduced/loss of dNTPase activity [37]
M385	Primary allosteric dGTP/GTP-binding site [49]	M385V	AGS [6, 43]	Subcellular localization affected, partially nuclear and cytosolic [37]; reduced HIV-1 restriction [37]; no oligomerization [37]; reduced/loss of dNTPase activity [37]
R442		R442X	AGS [6, 43]; uterine corpus endometrial carcinoma [ICGC]	Subcellular localization affected, partially nuclear and cytosolic [37, 43]; reduced LINE-1 and HIV-1 restriction [37, 99]; no oligomerization [37]; reduced/loss of dNTPase activity [37]
I448		I448T	AGS [43]; malignant lymphoma [ICGC]	Subcellular localization affected, partially nuclear and cytosolic [37]; reduced HIV-1 restriction [37]; reduced oligomerization [37]; reduced/loss of dNTPase activity [37]
R451	Primary allosteric dGTP/GTP-binding site [49, 101]	R451C	CLL [15]; skin melanoma [ICGC]	
		R451E	-	Reduced tetramer formation and loss of dNTPase activity [101, 104]
		R451H	CLL [16]	
		R451L	CLL [15]	
		R451P	Colon adenocarcinoma [26]	
		R451S	Liver hepatocellular carcinoma [ICGC]	
K484	Conserved residue, located on SAMHD1 surface [4]	K484T	Gastric cancer [4]	Reduced interaction with CtIP (leading to impaired DNA end resection after DNA damage), no influence on dNTPase activity [4]
D497		D497Y	Colon adenocarcinoma [26]	No dNTPase activity [26]
Y521	ssDNA binding [55]	Y521C	CLL [16]	
		Y521D	Myeloma [105]	
F545	ssDNA binding [55]	F545L	CLL [15]	
Q548	Second shell residue between activator and active site [104]	Q548A	-	Subcellular localization affected, partially nuclear and cytosolic [37]; reduced HIV-1 restriction [37]; normal oligomerization and dNTPase activity [37, 104]; reduced binding to ssDNA and ssRNA, reduced ssDNA cleavage [45]
		Q548X	AGS [6, 43]	Subcellular localization affected, partially nuclear and cytosolic [37, 43]; reduced LINE-1 and HIV-1 restriction [37, 99]; no oligomerization [37]; reduced/loss of dNTPase activity [37]

Selection of most interesting mutations that either have reported known functional consequences or a known involvement of the respective amino acid in structural integrity/cellular functions

In summary, it will be important to address how acquired *SAMHD1* mutations provide an advantage for cancerous cells and whether differences between tumor types are observable (for instance, hematological malignancies vs solid tumors). In general, it will be of great interest to understand how the different cellular roles of SAMHD1 (like dNTP homeostasis or involvement in DNA replication/DNA damage response (DDR)) are potentially disturbed through mutations, thereby likely driving oncogenesis.

## SAMHD1 mutations and their functional significance

SAMHD1 is involved in controlling absolute and relative cellular dNTP levels [2] and capable of degrading all four dNTPs [1, 27]. Therefore, functional mutations in SAMHD1 could lead to dNTP imbalances. Consequently, disturbed DNA replication fidelity along with spontaneous mutations could result in genomic instability, potentially promoting cancer development [28].

**Fig. 3:**
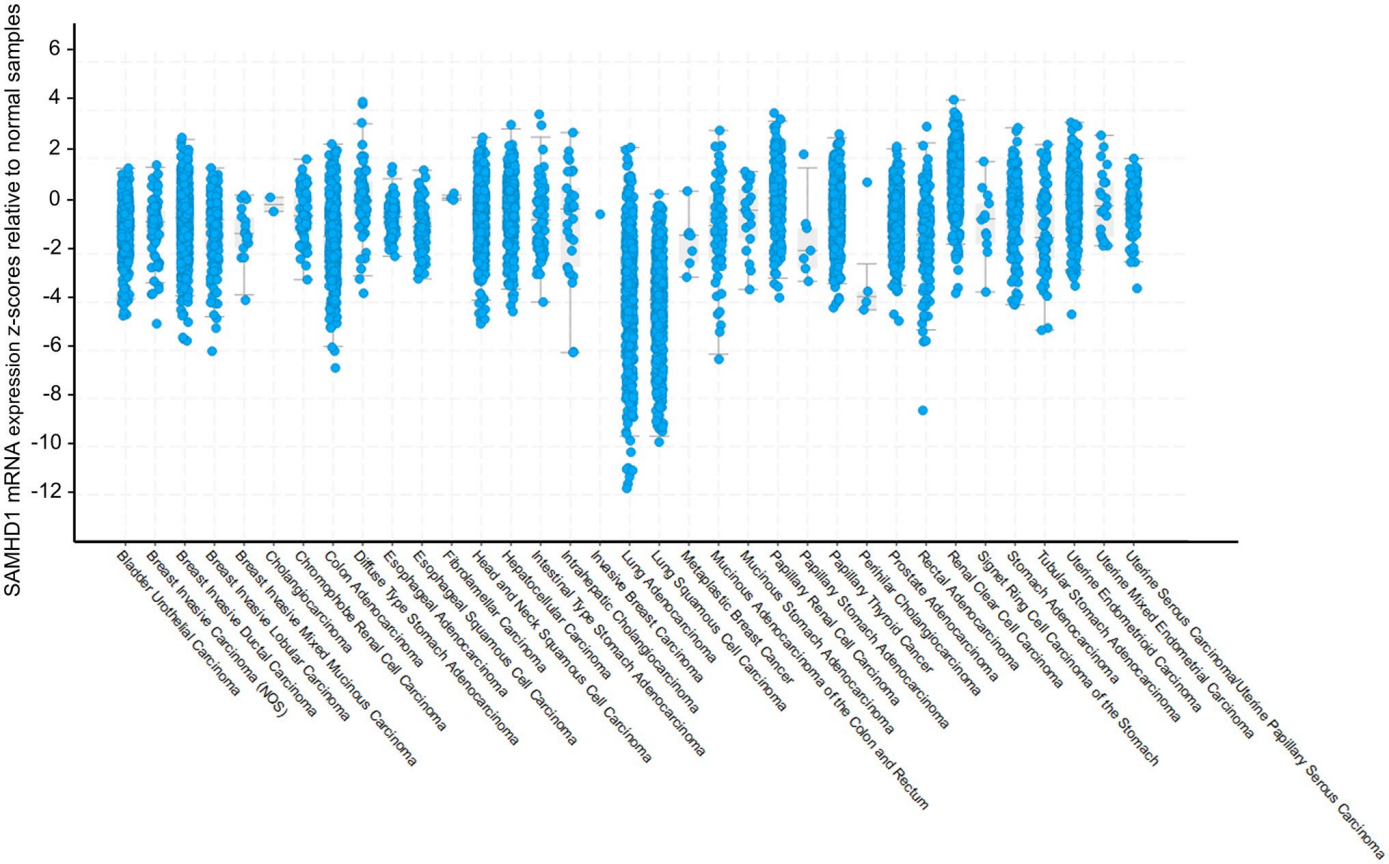
Expression of SAMHD1 in different cancer types Each point represents paired tumor/healthy samples and the relative difference of SAMHD1 expression between the two. The difference is represented as a *z*-score, which shows the number of standard deviations between the expression of SAMHD1 in the respective tumor sample and the mean expression of SAMHD1 in the healthy samples

The regulation of dNTP pools is important for cell cycle progression, as cycling cells need to carefully balance dNTP levels to ensure proper S phase completion and transition to mitosis [29]. During G1 phase, SAMHD1 maintains low dNTP levels. Only upon entering into S phase, the dNTPase activity of SAMHD1 is potentially downregulated through phosphorylation at residue T592 [30–32] and/or reduction of its protein level [2]. Cancer cells need to sustain chronic proliferation; therefore, they need high dNTP levels at all times [33]. This could be achieved by downregulating SAMHD1 expression (see Fig. 3) or through the acquisition of mutations in *SAMHD1* which abolish its dNTPase activity.

**Fig. 4:**
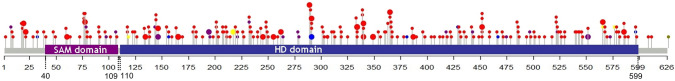
Graphical representation of the somatic mutation spectrum throughout the protein sequence of SAMHD1 In total, 177 coding mutations from ICGC cancer studies and other 53 mutations surveyed from the literature were visualized. The scale bar represents the length (amino acids) of the protein sequence. Each lollipop represents a somatic coding mutation. Lollipops are colored according to the consequence type: missense (red), frameshift (blue), stop-gain (purple), stop-lost (olive), deletion (yellow). The size of the lollipops represents the number of reported patients with the mutation. The lollipop diagram was created by using [106]. The domain structure is based on [100]. Supplemental Table 1 lists the 230 coding mutations inclusive cancer type and references

We visualized the expression of genes originating from the resource PanCancer Atlas [34] by using cbioportal [35, 36]. Interestingly, SAMHD1 displays a general downregulation in most cancer types, implying a correlation between cancer and SAMHD1 repression — with the strongest downregulation observed in lung cancers (Fig. 3). Next, we were interested how coding mutations are distributed throughout the protein sequence of SAMHD1. The lollipop diagram in Fig. 4 illustrates a graphical representation of the somatic mutation spectrum of SAMHD1. All 230 coding mutations seem to distribute relatively evenly throughout the whole protein sequence of SAMHD1 (Fig. 4, Supplemental Table 1), which, at first glance, does not allow us to draw any conclusions on certain protein domains that might be important for cancer development. This also suggests that not only the enzymatic activity of SAMHD1 might be responsible for tumor development.

In the next chapter, we will discuss the impact of cancer-associated mutations on specific functions of SAMHD1. As SAMHD1 is expressed to different levels in different tissues and cancers, the impact of SAMHD1 might vary depending on the tissue in question. Moreover, the diverse functions of SAMHD1 make it difficult to pinpoint the exact mechanisms how SAMHD1 contributes to tumor development. Furthermore, partial or complete loss of SAMHD1 expression could be caused by specific mutations. This can be observed in CLL where many patients show reduced or abolished SAMHD1 expression due to somatic mutations [15]. Therefore, in this review, our aim is to provide a detailed overview of the cancers that are affected by SAMHD1 and to summarize the reported mutations and corresponding functional consequences. The most interesting mutations that either have reported known functional consequences or a known involvement of the respective amino acid in structural integrity/cellular functions are listed in Table 1.

## Known and potential impact of cancer-associated mutations on SAMHD1 function

### Changes in structure/catalytic function

The functionality of SAMHD1’s dNTPase activity is dependent on the catalytic and the allosteric sites in the HD domain of SAMHD1 [1]. Therefore, mutations in this region of the protein can reduce or completely abrogate the dNTPase function. Goldstone et al. created a panel of catalytic and allosteric site mutants that show such effects. All reported positions (H206A/D207A, D311A, H233A, R164A, D137A, Q142A, R145Q) can be found mutated in cancer patients (see Table 1; Fig. 4, Suppl. Table 1). This indicates that mutations that interfere with dNTPase function might lead to dNTP pool imbalances in these patients that could cause genomic instability or a mutator phenotype. Additionally, mutations associated with colon adenocarcinoma (V133I, A338T, R266H, and D497Y) (Table 1) were shown to reduce the dNTPase activity of SAMHD1 (see previous section). The resulting dNTP pool imbalances caused an increase of mutation frequency, when combined with MMR deficiency [26]. Furthermore, some catalytic or allosteric site mutants are associated with AGS (residues H123; R143, R145, R164, H167, R333, M385, and Q548) (Table 1) and might therefore lead to the induction of IFN [6, 37]. These residues were also reported in cancer patients. Other catalytic and allosteric site mutations which can be found in cancer involve residues D137, Q142, H206, D207, H233, D311, R366, and R451 (Table 1).

The relationship of dNTP pool balance and genomic instability was primarily shown for the ribonucleotide reductase, the rate-limiting enzyme of the de novo dNTP pathway [33]. Consequently, also upregulation or downregulation of SAMHD1 expression or SAMHD1 mutations, which might alter its dNTPase activity, can contribute in a comparable manner [29, 38]. Dysregulated dNTP levels can be responsible for DNA replication stress and can affect DNA repair mechanisms [29]. Additionally, increased frequencies of DNA damage by dNTP pool dysregulation can induce IFN-stimulated genes, leading to chronic inflammation, a phenotype commonly observed in AGS patients [6, 38]. Another consequence of dNTP pool imbalances can be the increase of random genome-wide mutations, thus creating a mutator-phenotype, driving oncogenic transformation of pre-cancerous cells [39].

SAMHD1 has also been proposed to play a role during antibody class switch recombination [40]. During this process, non-homologous end joining (NHEJ) and microhomology-mediated end joining (MMEJ) are active and reported to be sensitive to dNTP imbalances caused by dNTPase-impaired SAMHD1. This could lead to nucleotide insertions at the recombination sites leading to genomic instability in B cells [41].

### Mislocalization

SAMHD1 is primarily localized in the nucleus due to the nuclear localization sequence ^11^KRPR^14^ [42]. Mutations that lead to changes in SAMHD1 localization have been identified in AGS patients and might therefore contribute to pathogenicity [43]. These mutations can also be found in cancer, especially CLL [15]. Mutations that are common in AGS and cancer are H123Y, R143H/C, R145X/Q, R164Q, G209C, I201N, R226H, M254I, R290C, D311E, R442X, and I448T (Table 1, also includes other amino acid changes at the same amino acid residue) [37, 43]. Interestingly, many of these AGS mutations were reported for CLL: R145X/Q, I201N, R290C, and M254I [15, 44]. Except for the D311 mutation, all of these mutants show mislocalization to the cytoplasm to different degrees [37]. This indicates that mislocalization of SAMHD1 might not only contribute to pathogenicity in AGS but also in certain cancer types, namely CLL. This phenomenon speaks for a role of SAMHD1 in replication and/or DNA damage response as nuclear localization might not be essential for dNTPase activity. However, mutations at R143, I201, R226, M254, D311, R442, and I448 were shown to have lost dNTPase activity [37, 45]. Therefore, future studies are needed to determine to which extent SAMHD1 mislocalization might influence or even obstruct its cellular activities. As these mutations are all reported AGS mutations, they might all lead to an upregulation of interferons as the typical AGS phenotype described by Rice et al. [46] although this remains to be conclusively validated.

### Dimer and tetramer formation

dNTPase-active SAMHD1 forms tetramers [47–49]. Phosphorylation at T592 downregulates the dNTPase function of SAMHD1 by interfering with protein tetramer stability [31, 32, 50]; however, conflicting results have been reported, de-coupling phosphorylation and dNTPase activity [51]. Phosphorylation of SAMHD1 is regulated during cell cycle progression [30, 31] and occurs in cycling cells during cell cycle phases that require high dNTP concentrations, i.e., during DNA replication in S phase. In terminally differentiated cells, SAMHD1 is usually not phosphorylated at residue T592 to maintain low dNTP pools [30]. It could be plausible that in certain cancers, SAMHD1 remains phosphorylated, as cancer cells are metabolically highly active with reduced or lost control mechanisms for cell growth. This can be observed in cultured cancer cell lines, e.g., cycling THP-1 cells [51]. Interestingly, so far, T592 mutations have not been reported to occur in cancers (see Table 1; Fig. 4, Suppl. Table 1). This could indicate that it is beneficial for cancers to maintain SAMHD1 phosphorylation. However, as it yet remains to be clarified how T592 phosphorylation is connected to tetramerisation and dNTPase function [52], one can only speculate on the influence of SAMHD1 T592 phosphorylation on cancer cells. In this context, SAMHD1 mutations that affect dimerization or tetramerization ability might play a role as well. Intriguingly, we could find two reported mutations (H364Q and H364K) deposited to ICGC which might affect SAMHD1 tetramerization [53].

### DNA replication and DNA end resection

Mutated SAMHD1 could not only contribute to genomic instability through an impaired dNTPase activity but also due to its role in end resection during DNA replication or DNA damage repair [4, 5]. In the absence of SAMHD1, end resection, a process necessary to resolve stalled replication forks and enable the repair of DNA double strand breaks, is not functioning properly [5]. Coquel et al. could show an interaction of SAMHD1 and MRE11 nuclease, stimulating the exonuclease function of this enzyme. This activity initiates DNA end resection and consequently downstream processes to activate DNA damage repair and replication fork restart [7]. Daddacha et al. made similar observations for CtIP, another enzyme involved in initiating the DNA end resection process, which also interacts with MRE11, linking SAMHD1 to the initiating events of homologous recombination (HR) [4]. In both reports, SAMHD1 facilitates recruitment of the factors to enable end resection.

Without end resection, cells are not able to replicate correctly, which could lead to accumulation of genomic mutations, thus contributing to genomic instability and possibly to mutation of proto-onco genes.

Involvement of SAMHD1 in end resection could provide a link to the reported ability of SAMHD1 to bind nucleic acids, like single-stranded DNA (ssDNA). This has been implicated in several publications [45, 54, 55], and although it is not clear whether SAMHD1 itself can act as a nuclease, it seems plausible that it can bind ssDNA and recruit nuclear endonucleases or exonucleases which can then degrade nucleic acids [55]. Interestingly, amino acid residues that were characterized by Seamon et al. to be involved in ssDNA binding overlap with cancer-associated mutations in the region between residue 360 and residue 545: Y360, H364, Y521, F545 (Fig. 4, Suppl. Table 1). These residues are not part of the active or allosteric site of SAMHD1. This might indicate that mutations in this region specifically alter ssDNA binding ability of SAMHD1, while other functions are not affected. It would be interesting to study these mutants with regard to their ability to promote DNA end resection by recruiting MRE11 exonuclease [5]. Another mutation that can be found in this protein region is the K484T mutant described by Daddacha et al. who have shown that this residue is important for the recruitment of CtIP to DNA damage sites to enable DNA end resection in HR [4]. Both these studies provide mechanistic insight how SAMHD1 plays a role in replication and DNA damage and how its ssDNA binding ability might be connected to this function. Interestingly it was also shown in both publications that the involvement of SAMHD1 in DNA end resection is dNTPase-independent. dNTPase-defective H206A/D207A [4] and K312A [5] were still able to induce the SAMHD1 wt phenotype in rescue experiments, while the partially dNTPase-active Y315A [45] mutant could not rescue the wt replication phenotype [5]. In contrast, the T592 phosphorylation seems to play a role in this regard, as the phosphoablative mutant T592A can no longer rescue the WT phenotype, while the phosphomimetic T592E mutant can [5]. This highlights that the T592 phosphosite might serve as a switch of function between controlling viral restriction and end resection, regulated during the cell cycle. During S-phase, phosphorylated SAMHD1 (at T592) supports the replication process, while it is rapidly dephosphorylated at T592 during mitotic exit and restrictive towards HIV-1. Mitotic exit might also mark the transition to its function as a dNTPase controlling the cellular dNTP pool [5, 30, 50].

Recently, another aspect emerged. At DNA replication-transcription conflict regions, R-loops (DNA:RNA hybrids) are formed and are associated with cancer development [56]. These are enriched in AGS patients with SAMHD1 deficiency and can cause replication stress and genome instability [57]. SAMHD1 was shown to be involved in resolving R-loops and it was suggested that this might also be connected to its ability to recruit MRE11 [57]. In this study, colorectal cancer-associated mutations (F59C, D207Y, R226H, T232M, K288T, and S247Y [58] (Fig. 4, Suppl. Table 1)) were investigated on their ability of regulating R-loops. Two of these mutations (F59C and T232M) showed increased transcription-replication conflicts when compared to SAMHD1 wt [57]. This provides another likely dNTPase-independent mechanism of how SAMHD1 could be involved in cancer.

### End joining

Recently, SAMHD1 was shown to be also involved in DNA end joining in NHEJ, a DNA repair pathway active throughout all cell cycle phases [59]. It was described that the dNTPase function of SAMHD1 is important for this repair pathway as a balanced dNTP pool is necessary to avoid nucleotide insertions at repair junctions, potentially linking SAMHD1 dysfunction to genomic instability and thereby promoting tumor development [60]. This was also observed during antibody class switching [40, 41]. This is especially intriguing, as SAMHD1 is frequently mutated in CLL, a form of B lymphocyte leukemia. This model provides an explanation for one specific subset of cells (B cells) and is of course not applicable for the majority of other tumors. In this context, the dNTPase defective K312A mutation leads to longer repair junctions harboring DNA insertions. In contrast, mutations K484T and K11A with intact dNTPase activity did not lead to longer DNA insertions [60]. A mutation at K484 is also found in the cancer data (Table 1; Fig. 4, Suppl. Table 1). This suggests that SAMHD1 might play different roles in different tumors, depending on tissue-specific factors, like expression, cell division rates, and specialization of a cell type.

### Innate signaling

Mutated SAMHD1 is strongly connected to inflammation. In the absence of functional SAMHD1, cells are not able to control upregulation of inflammatory signals. This can be observed in the hereditary autoimmune disease AGS, in which patients display chronically elevated IFN levels [6]. In this context, IFN stimulatory self-DNA has been implicated as a possible cause for the disease phenotype [61]. However, the exact reasons are not yet fully understood. On the one hand, SAMHD1 itself can act as a negative regulator of innate immunity [3]. On the other hand, SAMHD1 prevents aberrant DNA being dislocated into the cytoplasm by helping to resolve stalled replication forks [5]. As displayed in Fig. 1, SAMHD1 role in DNA repair might play an additional role in upregulation of innate immunity [8]. A similar concept might apply for cancer development as well. One could hypothesize that SAMHD1, if mutated or downregulated in cancer cells, would lead to accumulating self-DNA that might be sensed through the cGAS-STING pathway thus inducing a tumor-associated chronic inflammatory response [7, 62]. Therefore, SAMHD1 might be placed into the group of caretaker genes that protect cells from genomic instability [63] by reducing DNA damage and thereby potentially avoiding the induction of a strong immune response by self-DNA [38].

Tumors are often infiltrated by immune cells, both of the innate and adaptive arms, resembling inflammatory conditions [11]. Of course, a strong immune reaction is crucial to destroy tumors. Paradoxically, immune cells, particularly innate immune cells, can also contribute to neoplastic progression by providing bioactive molecules to the tumors which can facilitate tumor growth [11]. One inflammatory cytokine which has been linked to pro-tumor effects is the tissue necrosis factor α (TNF-α) when produced in the tumor microenvironment. TNF-α is produced by various cancers in small quantities and can promote cancer progression in various ways which are still under investigation [64, 65]. SAMHD1 was shown to be a negative regulator of innate immunity. Specifically, it has been linked to the Nf-κB pathway. SAMHD1 interacts with the Nf-κB inhibitor IκBα by blocking the phosphorylation and subsequent degradation of this inhibitor. Additionally, SAMHD1 inhibits IκB kinase ε (IKKε)-mediated IRF7 phosphorylation and by this reduces IFN-1 induction [3]. Therefore, missing or mutated SAMHD1 might contribute to a pro-tumor microenvironment, as the cells are not able to correctly downregulate inflammatory signals like TNF-α in the absence of SAMHD1 [3].

Also the cGAS-STING pathway, which is known to have important implications in anti-tumor immunity [62], can in some cancers promote inflammation-driven carcinogenesis, for example in brain metastasis [66] or skin cancer [67]. It is thought that DNA leakage into the cytoplasm triggers cGAS-STING-dependent production of pro-inflammatory cytokines like TNF-α [67].

There is little known about SAMHD1 mutations that influence its ability to downregulate the NF-κB pathway. It was shown that dNTPase activity is important for this function, as the dNTPase-defective SAMHD1 mutant H206R/D207N loses the ability to downregulate innate signaling in nondividing monocytic cells [68]. Therefore, it is possible that other SAMHD1 mutants with reduced dNTPase activity, e.g., R305A, D311A, K312A, and R143H (Table 1, Fig. 4, Suppl. Table 1) [45], also lose this function and contribute to chronic inflammation associated with the tumor microenvironment. Interestingly, for cycling cells, the dNTPase activity is not involved in downregulating innate immunity, highlighting that SAMHD1 functions seem to be highly dependent on cell cycle status and cell proliferation activity [3]. Also, the phosphorylation status at T592 does not seem to contribute to SAMHD1’s role in regulating the innate immune response [3].

Known AGS mutations can be found in various cancer types: R145X, R143C, R442X, R145Q, R611Q, R348C, D585N, P485S, A181T, R194X, R339C, R333H (overlap of ICGC cancer mutations and AGS mutations). Many of these mutations also have functional significance (see above and Table 1). As depicted in Fig. 1, SAMHD1 could be involved in different cellular processes, which lead to the upregulation of the IFN signaling, also independent of its dNTPase activity. It was shown that AGS patient-derived fibroblasts with either R290H, Q548X, or H167Y mutations display an altered dNTP pool leading to genomic instability and upregulation of IFN [38]. The R290 residue is also represented in the cancer data survey (Table 1, Fig. 4, Suppl. Table 1). In conclusion, the impact of SAMHD1 on innate signaling and perhaps on additional functions might contribute to inflammation-driven carcinogenesis.

## SAMHD1 as a potential tumor suppressor

Tumor suppressor genes are vital to regulate normal cell growth and proliferation. Therefore, their expression is repressed on the transcriptional level in various malignancies, for instance, through promoter methylation and/or histone modifications [69].

Initially, it was reported that *SAMHD1* mRNA and protein expression were reduced in peripheral blood mononuclear cells (PBMCs) obtained from patients with Sézary syndrome (SS), an aggressive subtype of cutaneous T-cell lymphoma (CTLC), compared to healthy donors [70]. In eight out of nine patient PBMCs examined, the *SAMHD1* promoter was highly methylated (up to 51-fold higher on average), whereas no promoter methylation could be observed in PBMCs from healthy donors [70]. Subsequent studies aiming to identify recurrently mutated/altered genes in SS patients found deletions or mutations in *SAMHD1* [71], potentially leading to altered *SAMHD1* expression, in > 10% of patients.

SAMHD1 downregulation on the mRNA and protein level, compared to CD4^+^ T-cells from healthy donors or monocytic THP-1 cells, was also observed in various CD4^+^ T-cell lines derived from leukemia and CTCL patients [72, 73]. Reduced SAMHD1 expression was achieved through transcriptional repression by promoter methylation [72], potentially in combination with microRNA-181 upregulation [73]. Specifically, an inverse correlation between miRNA-181b levels and SAMHD1 protein expression could be established [73]. Additionally, increased expression of all microRNA-181 family members (a-d) was detected in primary CD4^+^ T-cells from Sézary syndrome patients compared to healthy control cells, which was again associated with reduced SAMHD1 protein expression [73]. However, the exact contribution of both mechanisms to SAMHD1 downregulation, especially in CTCL patients (as, for instance, mRNA expression levels were in some patients reduced [70], while not in others [73]), would be interesting to explore in future studies. Nevertheless, to understand the impact of SAMHD1 downregulation in this cancer type, the CTCL-derived cell line HuT 78 (normally expressing low SAMHD1 levels) was stably transduced with full-length SAMHD1. As a result, reduced cell proliferation and colony formation, but higher levels of spontaneous and Fas ligand (FasL)-induced apoptosis were observed [74]. Therefore, it was proposed that SAMHD1 might act as a tumor suppressor in neoplastic T-cells partly by apoptosis induction [74].

As described, mutations in SAMHD1 were recurrently found in CLL patients leading to reduced mRNA expression and in most cases, but not all, almost complete loss of SAMHD1 protein expression compared to B-cells (mRNA) or PBMCs (protein) from healthy donors [15]. However, the exact mechanisms of mRNA/protein downregulation still need to be assessed in more detail. For instance, mutations could either interfere with proper transcription, induce non-sense mediated mRNA decay, or, in the end, could destabilize SAMHD1 (mutant) protein. Additionally, 12 out of 18 *SAMHD1*-mutated CLL patients showed abnormalities involving the *SAMHD1* locus, located on chromosome 20, including copy-neutral loss of heterozygosity (cnLOH), mosaic cnLOH, or even complete loss of the second allele [15].

In T-PLL, *SAMHD1* is not only recurrently mutated but deletions in the *SAMHD1* locus were additionally observed in two patients (2/14 patients, 14%), resulting in completely abolished SAMHD1 protein expression [22]. As already noted in some CLL patients, a strict correlation between lower *SAMHD1* mRNA levels and protein expression could also not be established for all T-PLL samples, again hinting at additional regulatory mechanisms. Additionally, the authors state that they could not detect hypermethylation of the SAMHD1 promoter in over 50 different T-PLL samples compared to healthy T-cells [22].

Acute lymphoblastic leukemia (ALL) is the most common cancer observed in children; due to improved treatment, survival rates have now increased from 10% in the 1960s to about 90% today [75]. Different subtypes of ALL might arise after malignant transformation of precursor cells from the B- or T-lymphoid lineage (B-ALL and T-ALL), characterized by specific alterations and gene expression patterns [75]. Rothenburger et al. were able to show specific differences in *SAMHD1* mRNA expression between T- and B-ALL: In cell lines derived from T-ALL, *SAMHD1* mRNA levels were significantly reduced compared to cell lines with B-ALL origin; the same mRNA expression pattern could also be detected in T- versus B-ALL blasts from 306 ALL patients [76]. Interestingly, a closer examination of *SAMHD1* mRNA levels in T- and B-ALL subgroups revealed further pronounced differences, which could be important for therapeutic outcome; for instance, *SAMHD1* mRNA levels were equally low in Philadelphia (Ph)-like B-ALL and T-ALL patient samples [76]. In T-ALL-derived cell lines, low *SAMHD1* mRNA levels also correlated with low protein expression [76]. In order to understand the marked differences in SAMHD1 expression among T- and B-ALL-derived cell lines, investigation of *SAMHD1* promoter methylation revealed that it was methylated in almost all T-ALL cell lines (10/11) tested, while being unmethylated in most B-ALL cell lines (13/15) [76]. Effectively, this observation suggests a lineage-specific regulation of *SAMHD1* promoter methylation.

Acute myeloid leukemia (AML) is a hematological cancer that is characterized by uncontrolled proliferation of myeloid precursor cells in the bone marrow and blood, ultimately interfering with normal production of blood cells [77]. *SAMHD1* mRNA expression levels differed widely in adult and pediatric AML patients [78]. As already observed in CTCL patients [70, 73], *SAMHD1* mRNA expression negatively correlated with promoter methylation and levels of miRNA-181a [78]. With this, Herold et al. could define two distinct groups of AML patients (SAMHD1-low and -high expression) that respond differently to treatment with high-dose cytarabine (cytosine arabinoside, ara-C) consolidation therapy [78] (see last section in this review). Additionally, it was reported that *SAMHD1* mRNA expression was downregulated in bone marrow samples of AML patients compared to a non-AML patient group [79]. *SAMHD1* mRNA expression was not correlated with other downregulated apoptotic genes (*BAD*, *BAX*, *BAK1*, *XIAP*, and *BIRC2*) known to be relevant in AML pathogenesis. In this (small) AML cohort, however, low *SAMHD1* mRNA expression was not associated with worse prognostic outcome (e.g., represented by white blood cell count or blast percentage) or reduced OS [79]. Nevertheless, a potential role of SAMHD1 as a tumor suppressor in AML still needs further investigation, as low SAMHD1 levels in AML bone marrow samples might indicate its role in leukemia induction. Investigating the potential involvement of different SAMHD1 functions, e.g., dNTPase, regulation of DNA replication/DDR, or inflammatory signaling in AML development and progression would be of great interest.

Only recently, SAMHD1 protein expression was assessed by immunohistochemistry in Hodgkin lymphoma (HL): Staining for SAMHD1 was mainly observed in the nucleus of Hodgkin and Reed-Sternberg (HRS) cells, which are the distinct neoplastic cells derived from mature B-cells found in HL. In total, only 31% (48/154) of HL samples evaluated were categorized as positive for SAMHD1 protein expression [80], implicating a potential downregulation of SAMHD1 in HL. The authors could also correlate SAMHD1 expression with clinical outcome in 125 HL patients: Here, positive SAMHD1 expression in HRS cells was linked to inferior freedom from progression (FFP; 51% vs 70%), disease-specific survival (DSS; 72% vs 92%), and 10-year OS (OS; 69% vs 86%) compared to HL patients with SAMHD1-low/negative HRS cells [80]. Therefore, SAMHD1 was suggested to be used as an independent marker for HL prognosis. It will be of great interest in future studies to determine how higher SAMHD1 expression can lead to poorer clinical outcome in HL.

Additionally, SAMHD1 expression seems to be downregulated, both on the mRNA and protein level, in malignant tissue from five lung adenocarcinoma (LAC) patients compared to the surrounding unaffected lung tissue [81] (see also Fig. 3). Again, the authors could correlate high *SAMHD1* promoter methylation with lower SAMHD1 levels in lung adenocarcinoma [81]. To support the observations made in LAC patients, treatment of lung carcinoma-derived cell lines (A549, H1299) with the methyltransferase inhibitor 5-Aza-dC (decitabine) led to increased *SAMHD1* mRNA and protein levels [81]. Combination of 5-Aza-dC with the histone deacetylase inhibitor trichostatin A (TSA) further increased *SAMHD1* mRNA induction, indicating that several epigenetic mechanisms control SAMHD1 expression [81]. Mechanistically, overexpression of exogenous SAMDH1 in A549 cells led to reduced dNTP levels and cell proliferation compared to control cells [81]. Therefore, high SAMHD1 expression might confer a growth disadvantage for LAC cells.

In a subsequent study, *SAMHD1* mRNA expression in tumor and adjacent healthy tissue was assessed in a larger cohort of 238 non-small lung cancer (NSLC) patients: Again, expression of *SAMHD1* mRNA was significantly reduced in tumor compared to healthy specimens [82]. Interestingly, lower *SAMHD1* mRNA expression correlated with a more aggressive, metastatic course of disease in patients [82]. To further understand SAMHD1’s involvement in lung cancer development and progression, A549 cells were stably transduced with SAMHD1 and reduced cell proliferation, colony formation, and apoptosis induction could be observed [82]. In line with the clinical data, SAMHD1 overexpression reduced A549 cell migration and invasion [82]. However, a potential negative regulation of STING by SAMHD1 and a resulting suppression of LAC progression, suggested by Wu et al., urgently need further experimental clarification [82].

According to our analysis, SAMHD1 expression can differ considerably between patients in lung adenocarcinoma (Fig. 3); therefore, it will be important to determine the exact impact of SAMHD1 downregulation in lung adenocarcinoma development and/or progression in future studies, especially the underlying molecular mechanisms.

The incidence of cutaneous melanoma (or skin cutaneous melanoma, SKCM), one of the most aggressive forms of skin cancer due to its potential to metastasize, is increasing annually and displaying high levels of somatic genetic alterations [83]. Using changes in DNA methylation profiles and copy number variations (CNVs) observed in SKCM, Chen et al. were able to define four distinct SKCM subtypes (iC1–iC4) that differed in their prognostic outcome — with the iC3 subtype showing the poorest OS [84]. Having a closer look at genes with distinct differences between subtypes (in terms of methylation status, CNV, and mRNA expression) revealed that 146 genes were actually correlated with prognosis [84]. Indeed, decreased mRNA expression and hypermethylation of *SAMHD1* (along with *GBP5*, *CD8A*, and *KIAA0040*) were associated with reduced survival rate of patients clustered in the iC3 compared to the iC1 subtype [84].

A first analysis of primary breast cancer samples indicated that SAMHD1 protein expression might be reduced or even absent in approximately 50% of cases [15]. However, SAMHD1 protein expression was only compared to THP-1 and SupT1 cells, not to matched healthy breast tissue, and the reason for lower SAMHD1 expression (for instance, promoter methylation and/or detrimental mutations) will need further clarification. Additionally, it would be of great interest in further studies of breast cancer samples to discriminate between different subtypes and correlate whether differing SAMHD1 expression has an impact on disease prognosis.

Cancer-derived cell lines can be a viable tool to understand the molecular mechanisms leading to uncontrolled cell growth observed in malignant diseases. Specifically, it will be important to investigate the consequences of SAMHD1 deregulation (whether it is due to mutations or mRNA/protein downregulation) for cellular dNTP metabolism, DNA replication, and/or DDR which is necessary to evaluate SAMHD1’s role in cancer development and progression. The effect of SAMHD1 protein reduction or absence on cell proliferation, however, seems to differ depending on the cell type or cellular context. In a first study, reduction of SAMHD1 through RNA interference (RNAi) in immortalized cycling lung fibroblasts resulted in reduced cell growth, as G_1_/S transition during cell cycle-progression seemed to be disturbed. Consequently, cells accumulated in G_1_ phase, while the amount of S phase cells was reduced; however, no concomitant decline in dNTP concentrations could be observed [2]. Using SAMHD1-deficient primary fibroblasts from two different AGS patients, Kretschmer et al. could measure significantly upregulated dNTP levels, while the AGS fibroblasts also proliferated slower compared to healthy control cells [38]. In addition, AGS fibroblasts showed an overall decrease in genomic integrity and upregulation of several DDR genes [38].

CRISPR/Cas9-mediated knock-out (KO) of SAMHD1 in monocytic, AML-derived THP-1 cells resulted in increased cellular dNTP levels and an enrichment in G_1_/G_0_ phase-cells, while a reduction only in the G_2_/M population occurred [10]. In contrast to human fibroblasts [2, 38], these changes led to increased proliferation of THP-1_KO SAMHD1 compared to control cells [10].

By now, several studies also investigated the influence of SAMHD1 overexpression on proliferation in cancer-derived cell lines: In the cervical carcinoma-derived HeLa cells [15] and the lung cancer-derived cell line A549 [81], SAMHD1 overexpression led to reduced cell proliferation [15, 81] and a decrease in cellular dNTP levels [81]. Similar results were obtained using the CTCL-derived cell line HuT 78: SAMHD1 overexpression reduced dNTP concentration and cell proliferation, while apoptosis rates in these cells were elevated [74]. However, Herold et al. could not confirm the impact of SAMHD1 absence (in THP-1 cells) or overexpression (in HuT 78 cells) on cell proliferation [85]. Nevertheless, these conflicting results again highlight the importance of understanding the interplay of deregulated dNTP pools, DDR induction, and, ultimately, control of cellular proliferation due to changes in SAMHD1 activity. The contribution of SAMHD1’s different cellular functions will be important to elucidate — specifically, in various physiological and malignant cellular environments and particularly in primary cells.

In summary, SAMHD1’s potential role as a tumor suppressor is underlined by various studies and our own analyses (Fig. 3) that show its downregulation or deregulation through mutations in malignant diseases. Additionally, in many cancers, high expression levels of SAMHD1 are associated with a more favorable outcome (Table 2). Ultimately, SAMHD1 is required to balance cellular dNTP concentrations and/or regulate DNA repair/replication and, therefore, avoid mutagenic conditions favorable for cancer development and progression.

**Table 2:** Prognostic association of high SAMHD1 expression level in 17 major cancer types

**P-values**	**Prognosis**	**Cancer type**
0.007	Favorable	Cervical cancer
0.016	Favorable	Colorectal cancer
0.019	Favorable	Head and neck cancer
0.032	Favorable	Thyroid cancer
0.038	Favorable	Endometrial cancer
0.041	Favorable	Lung cancer
0.104	Favorable	Prostate cancer
0.109	Favorable	Breast cancer
0.128	Favorable	Glioma
0.203	Favorable	Melanoma
0.001	Unfavorable	Renal cancer
0.027	Unfavorable	Urothelial cancer
0.091	Unfavorable	Testis cancer
0.144	Unfavorable	Stomach cancer
0.188	Unfavorable	Pancreatic cancer
0.215	Unfavorable	Liver cancer
0.363	Unfavorable	Ovarian cancer

Results of the study [107] were used to compile the table depicting the prognostic association of SAMHD1 expression level in reported cancers. In the study, the transcriptomes of 17 major cancer types were analyzed with respect to clinical outcome to explore the prognostic role of each protein-coding gene in each cancer type. For each gene and cancer type, the patient cohort was stratified into two groups based on individual expression levels. The data included transcript expression levels summarized per gene (fragments per kilobase of exon per million mapped reads — FPKMs) in 7932 samples from 17 different cancer types. To choose the best FPKM cutoffs for grouping the patients for SAMHD1 most significantly, all FPKM values from the 20th to 80th percentiles were used here in testing for differences in the survival outcomes of the groups, and the FPKM value yielding the lowest log-rank *P* value was selected. Two types of prognostic genes affecting patient survival were defined: (i) SAMHD1 as an unfavorable prognostic gene, for which higher expression was correlated with a poor patient survival outcome, and (ii) SAMHD1 as a favorable prognostic gene, for which higher expression was correlated with a longer survival

## SAMHD1 as a potential driver gene

During the last 15 years, the genomic/mutational landscape of different human malignancies was extensively explored using large-scale sequencing efforts. Various genetic aberrations (from somatic mutations, to chromosomal as well as epigenetic changes) were identified that could potentially contribute to cancer development. Indeed, some genes can confer a selective growth advantage to cancerous cells when mutated, therefore, can promote tumorigenesis and/or cancer progression (= driver genes). These driver genes can be categorized to regulate three crucial cellular processes, namely cell fate, survival, and genome integrity [86, 87].

First indications that *SAMHD1* might act as a driver gene were provided by a study from Schuh et al. tracking the clonal evolution in individual CLL patients during treatment [14]. In one patient, a somatic mutation in *SAMHD1* was already detected in a founder subclone, suggesting it to be a potentially driving event in CLL [14]. Furthermore, subsequent whole-exome sequencing (WES) of CLL and germline DNA samples revealed that *SAMHD1* was recurrently mutated in 2.5% (4/160) of CLL patients [44]. Although *SAMDH1* mutations were found at lower frequencies compared to established CLL driver genes (like *MYD88*, *TP53*, or *ATM*), it was still classified as a potential driving cause in CLL [44]. All 20 potential driver genes are involved in seven specific signaling pathways (including DNA repair, cell cycle-control, and inflammatory pathways) known to be important in CLL [44]. As of today, SAMHD1’s involvement in several of these cellular processes could be shown (see section SAMHD1 function in this review), thereby directly linking SAMHD1 to these very same pathways involved in CLL development. However, SAMHD1’s role and impact as a potential driver gene might differ depending on CLL stage (for instance, before therapy and relapse after therapy). Using WES and deep resequencing, Amin et al. were able to examine paired samples (pre- and post-treatment) of 61 relapsed CLL patients, in order to uncover mutations in genes potentially driving CLL relapse [16]. First, *SAMHD1* mutations were recurrently identified in 9.8% (6/61) of rCLL patients. In 53 paired DNA samples from patients before and after therapy, only mutations in *TP53* and, to a lower extent, *SAMHD1* were identified to be commonly enriched post-treatment: In 7.5% of rCLL patients (4/53), a marked enrichment of mutated *SAMHD1* from already existing subclones could be detected at relapse [16]. These observations indicate that mutations in *SAMHD1* are rather important to drive CLL relapse and/or impede CLL chemo-immunotherapy, than for early events during CLL onset/progression.

Additionally, *SAMHD1* was only recently identified as a potential novel driver gene in MCL, with missense or deletion mutations in *SAMHD1* being present in 10% of patients from the analyzed MCL cohort [88]. Of note, MCL can be subdivided into two molecular subgroups, namely conventional MCL (cMCL) and non-nodal MCL (nnMCL), which differ in their cellular origin, genetic features, and clinical outcome [24]. Compared to nnMCL, cMCL is characterized by a higher number of genetic aberrations (including structural variations and copy number alterations), although different mutation types occur at almost the same rate in both subtypes [88]. Nevertheless, mutations in *SAMHD1* were only identified in cMCL patients; however, the present cohort comprised more cMCL (74%) compared to nnMCL (26%) cases, thereby not precluding that *SAMHD1* mutations can also occur in nnMCL patients [88]. It will be interesting to explore the contribution of *SAMHD1* mutations on the molecular level to MCL pathogenesis in future studies.

*SAMHD1* seems not to be a highly mutated driver gene compared to known cancer drivers like *TP53* (= tumor suppressor) or *KRAS* (= oncogene) [86, 89]. In some types of cancer, it could therefore be more difficult to identify *SAMHD1* as a potential driver gene merely based on mutation frequency [86]. However, *SAMHD1* was not recognized as a major driver gene in several studies using different methods which were not solely based on mutation frequency (e.g., [89, 90]).

Nevertheless, the pattern of cancer-related mutations that are found throughout the sequence of *SAMHD1* (Fig. 4) together with the fact of its downregulation in several tumors (Fig. 3) rather indicates that SAMHD1 might act as a tumor suppressor. Therefore, future studies are needed to pinpoint which mutations in *SAMHD1* might be true driver gene mutations (e.g., truncating, thereby inactivating, SAMHD1 or missense mutations influencing specific functions, like its dNTPase activity), not only passenger mutations that do not confer a selective growth advantage. Consequently, it is important to understand in which specific types of cancer and/or disease stages *SAMHD1* mutations or expression level variations (e.g., through epigenetic changes) would promote malignant initiation and/or progression. With this knowledge, it will be possible to improve therapeutic efforts for individual malignancies or specific patients, as the genomic landscape of mutated (driver) genes can differ in each case appreciably.

Another interesting aspect has been discussed by Rentoft et al. [26]. Cancer-related, dNTPase-inactivating mutations in *SAMHD1* might act in concert with other genetic defects (e.g., in DDR pathways like MMR) to promote a so-called mutator phenotype [26]. In this case, dNTP pool imbalances through SAMHD1 dysfunction in combination with defects in DNA repair/proofreading pathways could, potentially, lead to genomic instability, thereby favoring malignant transformation. SAMHD1 was proposed to rather act as a “mini driver” [26, 91], meaning that mutations in SAMHD1 might only slightly increase evolutionary fitness of tumor cells. However, in combination with mutations in other “mini-driver genes,” the effects of SAMHD1 mutation might add up and, ultimately, could lead to a growth advantage equivalent to major driver gene mutations [91], worthwhile to investigate. Therefore, future studies are needed to characterize SAMHD1-mutated cancer types and accompanying mutations in other, potential driver genes.

## Outlook: SAMHD1 as a potential biomarker for treatment/clinical trials

As described, increasing evidence points towards a tumor suppressive role of SAMHD1 in different cancer types. However, several studies have shown by now that SAMHD1, due to its dNTPase activity, can also have a significant (negative) impact on the efficacy of nucleoside-based chemotherapies: For instance, different steps of AML therapy are often built around the nucleoside analog cytarabine (ara-C) which is converted intracellularly into the cytotoxic metabolite ara-C triphosphate (ara-CTP) [92]. SAMHD1 is able to degrade ara-CTP in vitro [78, 93, 94] and reduce its concentrations in cells like patient-derived AML blasts [78, 94], thereby posing a significant barrier to effective ara-C-based treatment. Indeed, SAMHD1 expression levels correlated with the effectiveness of ara-C therapy in different AML patient cohorts [78, 94]. High SAMHD1 expression correlated with a poorer response to ara-C-based AML induction [94], which was not observed in another study [78], as well as to consolidation therapy [78]. Therefore, SAMHD1 could be used as a marker to predict the outcome of ara-C-based regimens at different therapy stages [78, 94]. Additional studies are needed to further delineate how SAMHD1’s different functions (namely, being a barrier to ara-C-based therapies versus its potential tumor suppressive role) might influence each other, in order to predict the clinical response to ara-C of individual patients more precisely.

Additionally, SAMHD1 is able to hydrolyze several active triphosphate (TP) forms of nucleoside analogs used for anti-cancer therapies. Initial studies could show that SAMHD1 is able to degrade the TP form of clofarabine, which is used to treat pediatric ALL, at comparable rate to normal dNTPs [50, 95]. By comparing cytotoxicity of different nucleoside analogs in THP-1 cells and SAMHD1 knock-out THP1 cells, the resulting TP forms of vidarabine, nelarabine, fludarabine, trifluridine, and decitabine could be identified as potential SAMHD1 substrates [96], confirmed in structural studies [97]. Indeed, high SAMHD1 expression correlated with a poorer clinical response of AML patients to decitabine [98]. SAMHD1’s ability to degrade antimetabolites used in cancer therapy provides a rationale to directly alter the expression of SAMHD1, e.g., through Vpx-induced degradation [78, 94] or inhibit its activity using specific compounds to improve the efficacy of nucleoside-based chemotherapies.

Can SAMHD1 be regarded as a curse or cure for cancer? On the one hand, SAMHD1 appears to be a tumor suppressor, high expression correlates with a beneficial prognosis for many (but not all) cancers (Table 2), and mutations can have harmful effects (Table 1). However, SAMHD1 expression also interferes with nucleoside-based chemotherapeutics. One option would be to stratify patients based on their expression and mutation status to enable effective treatment. In conclusion, SAMHD1 can be both, curse and cure.

## Supplementary Information

Below is the link to the electronic supplementary material.

Supplementary file1 (XLSX 24 KB)
